# Videothoracoscopic Left Upper Lobectomy for Lung Cancer in a Case of Situs Inversus Totalis

**DOI:** 10.7759/cureus.16217

**Published:** 2021-07-06

**Authors:** Ali Celik, Muhammet Sayan

**Affiliations:** 1 Thoracic Surgery, Gazi University, Ankara, TUR

**Keywords:** lung cancer., situs inversus totalis (sit), video-assisted thoracoscopic surgery (vats), pulmonary lobectomy, mediastinal lymph nodes

## Abstract

The coexistence of situs inversus totalis (SIT) and lung cancer is exceedingly rare; therefore, only a single case report about this exists in the literature. Recent technological advancements in endoscopic surgery have allowed the execution of videothoracoscopic lung resection in these cases. However, the distinct placement of thoracic structures, bronchovascular anatomy, and additional anomalies in SIT should be investigated using bronchoscopy and contrast-enhanced computed tomography (CT). We present a case report of a videothoracoscopic left upper lobectomy with mediastinal lymph node dissection in a 50-year-old female patient with SIT.

## Introduction

Situs inversus totalis (SIT) is a rare autosomal recessive disease (reported incidence is 1 in 8000-20000 cases) in which thoracic and abdominal viscera of the left and right side are mutually displaced in the sagittal plane and placed as a mirror image of their normal positions [1-2]. Furthermore, the coexistence of SIT and lung cancer is exceedingly rare; therefore, it is only present as a case report in the English literature [3]. In such a case, like other non-small cell lung cancers (NSCLC), the optimal treatment option is surgical resection if the patient’s medical condition and stage of the cancer favor. Recently, few case reports have been published that describe lung resection by video-assisted thoracic surgery (VATS) in patients with SIT [2-5]. We present a case report of a patient with SIT undergoing VATS lobectomy for the left upper lobe with mediastinal lymph node dissection for an acinar-type adenocarcinoma, along with a discussion of the surgical technique used, and the intraoperative anatomy of the patient.

## Case presentation

**Figure 1:**
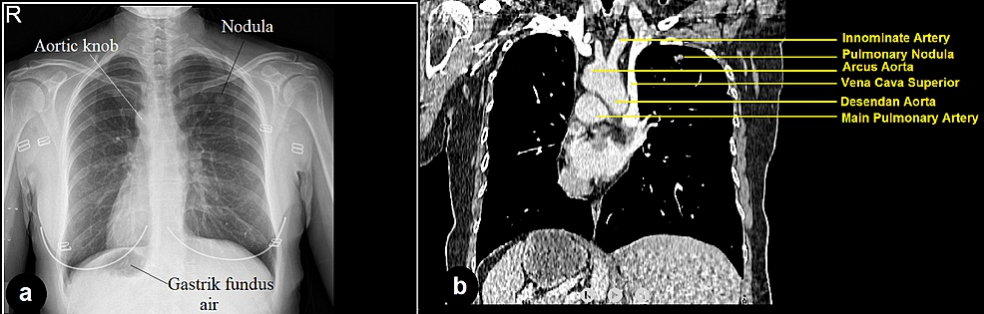
Chest X-ray showing situs inversus totalis and a solitary pulmonary nodule located in left upper zone; b: Thorax CT shows mirror image of vascular structures.

A 50-year-old female patient was referred to the department of thoracic surgery, Gazi University for a solitary pulmonary nodule in the left upper lobe detected on computed tomography (CT) of the thorax, which was performed for suspected coronavirus disease 2019 (COVID-19)-related pneumonia. She did not have a history of smoking or any other cardiopulmonary symptoms; the physical examination and routine laboratory tests were non-remarkable. A solitary pulmonary nodule located in the left upper zone and dextrocardia were detected on the initial chest X-ray, following which CT-thorax was done, which revealed SIT and an irregularly shaped pulmonary nodule (15 x 14 mm in diameter) in the left upper lobe (Figures 1a-1b). On positron emission tomography-computed tomography (PET-CT) scan, the 18f-FDG uptake in the nodule showed a pathological increase (SUV max: 10).

**Figure 2:**
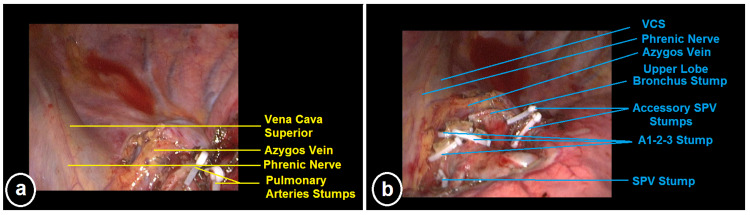
Intraoperative views of the patient (a-b). A1: Apical segmental pulmonary artery branch, A2: Posterior segmental pulmonary artery branch, A3: Anterior segmental pulmonary artery branch. SPV: Superior pulmonary vein, VCS: Vena cava superior.

A decision for surgical wedge resection of the nodule, followed by examination of its frozen section, was made; additionally, if required, a lobectomy with mediastinal lymph node dissection was also planned. Informed consent for the procedure, including publication of the radiological images, intraoperative photographs, and videos, was obtained from the patient, and she was taken to the operating room. Following the double-lumen intubation, her left hemithorax was explored in the lateral decubitus position using the bi-portal VATS method. We detected a mirror image of the right hemithorax in the left hemithorax, i.e., the right-sided anatomical structures, such as the three-lobed lung, superior vena cava, and azygos vein, were present on the left side. Additionally, the fissure of the superior segment of the left lower lobe was separate. The pulmonary artery branches of the left upper lobe included a two-branched truncus anterior (apical-posterior), a two-branched posterior artery, and a separate single-branched anterior segmental artery. Likewise, the superior pulmonary vein was formed from the upper and middle lobe veins. IIn addition, there were accessory pulmonary vein branches arising from the lower lobe vein and draining the upper lobe (Figures 2a-2b).

After identifying the relevant anatomical structures, the nodule was excised by wedge resection, and a frozen section was prepared for examination. The frozen section examination reported the nodule to be NSCLC; consequently, a left upper lobectomy with mediastinal lymph node dissection (intralobar, hilar, paratracheal, and subcarinal) was done. During hilar dissection, the azygos vein was injured, so it was divided with a vascular stapler to facilitate exposure of the upper lobe bronchus and control bleeding. Her postoperative follow-up was uneventful, and she was discharged on the fifth postoperative day. The final histopathological report described the nodule as acinar-type adenocarcinoma, and the pathological stage of the tumor was classified as T1aN0M0/Stage-IA1 according to the 8th TNM classification.

## Discussion

In this case report, we described the VATS lobectomy and mediastinal lymph node dissection procedure performed in a patient with combination of SIT and lung cancer, which is an exceptional entity. Baruah et al. (1952) published the first report on lung resection for a small-cell carcinoma in a patient with SIT [1]. Although advances in surgical techniques over the years have led to the application of minimally invasive surgical methods for such patients, there is a paucity of literature elucidating the role of VATS anatomical lung resection in coexisting NSCLC and SIT; we have reviewed these papers to compare them with our case report.

Previous studies have reported patients’ ages ranging from 48 to 74 years with an equal distribution for both genders (Table 1). Also, the tumors were predominantly located on the right side. Matsui and Gonzalez-Rivas reported synchronous lung cancers detected in the right upper and lower lobes [6,7]. Our patient was a 50-year-old woman with a lesion located in the upper lobe of the left lung. The majority of patients in the earlier papers were asymptomatic. However, in the case reported by Juma et al., the patient had symptoms of cough, chest pain, and hemoptysis. Hemoptysis and other symptoms may not be related to situs inversus per se, but may be representative of proximity of the mass with important bronchovascular structures [4]. Notably, the nodule was located peripherally in our patient, and she had no related symptoms. Furthermore, no perioperative and postoperative complications were reported in the previous case reports; although our patient’s left azygos vein was injured during hilar dissection, hemostasis was achieved without any problems, and no additional complications occurred both peri- and post-operatively. Another important finding during the literature review was that the tri-portal VATS technique was most frequently used in these patients. We used the bi-portal technique, like Yoshida et al., who performed a VATS right lower lobectomy via the bi-portal technique [5]. Incisions were made over the 7th intercostal space for the camera and the 4th intercostal space for the instruments. Gonzalez-Rivas et al. reported VATS segmentectomy using the uni-portal method [7]. 

**Table 1:** Available literature related to videothoracoscopic lobectomy for lung cancer in patients with situs inversus totalis. D.O.H: Duration of hospitalization, LUL: Left upper lobectomy, mm: Millimeter, RLL: Right lower lobectomy, RS1+S2+S9: Right apicoposterior and laterobasal segmentectomy, RS1+S2+S6: Right apicoposterior and lower lobe superior segmentectomy, RUL: Right upper lobectomy, SQCC: Squamous cell carcinoma.

Author	Year	Age	Sex	Location	Diameter	Symptoms	Operation	Complications	Histopathology	D.O.H	Technique
Kanayama et al. [2]	2018	61	F	Left Upper Lobe	25x12 mm	Asymptomatic	LUL	Nil	Adenocarcinoma	9	Tri-portal
Ye et al. [3]	2017	47	M	Right Upper Lobe	25 mm	Asymptomatic	RUL	Nil	Adenocarcinoma	5	Tri-portal
Juma et al. [4]	2017	62	M	Right Lower Lobe	32x31 mm	Cough, Chest Pain Hemoptysis	RLL	Nil	Adenocarcinoma	?	Tri-portal
Yoshida et al. [5]	2013	74	M	Right Lower Lobe	21x15 mm	Asymptomatic	RLL	Nil	Adenocarcinoma	?	Bi-portal
Matsui et al. [6]	2018	68	M	Right Upper/Lower Lobe.	15/19 mm	Asymptomatic	RS1+S2+S9	Nil	SQCC	8	Tri-portal
Gonzalez-Rivas et al. [7]	2018	48	F	Right Upper/Lower Lobe	10/8 mm	Asymptomatic	RS1+S2+S6	Nil	Adenocarcinoma	4	Uni-portal
Our Case	2021	50	F	Left Upper Lobe	15x14 mm	Asymptomatic	LUL	Azygos vein injury	Adenocarcinoma	5	Bi-portal

Strikingly, histopathological results for almost all cases with SIT and lung cancer were reported as adenocarcinoma, like our patient (Table 1). However, Matsui et al. described a case of synchronous squamous cell carcinoma treated by VATS segmentectomy in a patient with SIT [6]. Lastly, the duration of hospitalization reported in the available case reports is 4-9 days; comparably, our patient was discharged on the fifth postoperative day without any postoperative complications.

## Conclusions

VATS lobectomy and mediastinal lymph node dissection procedures can be safely performed for NSCLC in patients with SIT. In these patients, attention should be paid to the symmetrical placement of anatomical structures in the thorax. In addition, unexpected courses may be encountered in the pulmonary arteries, veins, and bronchi intraoperatively. So, preoperative thorax CT-angiography and bronchoscopy should be performed to prevent intraoperative complications due to additional bronchovascular abnormalities.
